# Cdk4 functions in multiple cell types to control *Drosophila* intestinal stem cell proliferation and differentiation

**DOI:** 10.1242/bio.016584

**Published:** 2016-02-15

**Authors:** Mojca Adlesic, Christian Frei, Ian J. Frew

**Affiliations:** 1 Institute of Physiology and Zurich Center for Integrative Human Physiology, University of Zurich, Zurich 8057, Switzerland; 2 Institute of Cell Biology, ETH Zurich, Zurich 8093, Switzerland; 3 Institute of Biomedical Engineering, ETH Zurich, Zurich 8092, Switzerland

**Keywords:** Enterocyte, Intestinal stem cell, Homeostasis, Cdk4, Niche

## Abstract

The proliferation of intestinal stem cells (ISCs) and differentiation of enteroblasts to form mature enteroendocrine cells and enterocytes in the *Drosophila* intestinal epithelium must be tightly regulated to maintain homeostasis. We show that genetic modulation of CyclinD/Cdk4 activity or mTOR-dependent signalling cell-autonomously regulates enterocyte growth, which influences ISC proliferation and enteroblast differentiation. Increased enterocyte growth results in higher numbers of ISCs and defective enterocyte growth reduces ISC abundance and proliferation in the midgut. Adult midguts deficient for Cdk4 show severe disruption of intestinal homeostasis characterised by decreased ISC self-renewal, enteroblast differentiation defects and low enteroendocrine cell and enterocyte numbers. The ISC/enteroblast phenotypes result from a combination of cell autonomous and non-autonomous requirements for Cdk4 function. One non-autonomous consequence of Cdk4-dependent deficient enterocyte growth is high expression of Delta in ISCs and Delta retention in enteroblasts. We postulate that aberrant activation of the Delta–Notch pathway is a possible partial cause of lost ISC stemness. These results support the idea that enterocytes contribute to a putative stem cell niche that maintains intestinal homeostasis in the *Drosophila* anterior midgut.

## Abstract (precis)

**Summary:** We identify that the growth status of absorptive enterocyte cells in the *Drosophila* intestine controls the proliferation and differentiation of stem and progenitor cells, thereby controlling organ homeostasis.

## INTRODUCTION

The *Drosophila* intestinal epithelium lines the lumen of the gut and consists of polyploid, absorptive enterocytes interspersed with small, diploid and basally embedded intestinal stem cells (ISCs), hormone secreting enteroendocrine cells and more apically located enteroblasts (Micchelli and Perrimon, 2006; Ohlstein and Spradling, 2006). The fly intestine is sub-divided into several anatomical regions; the foregut, midgut and hindgut, with each segment maintaining distinct functions. The longest part of the intestine is the midgut, which functions in nutrient breakdown and absorption and acts as a barrier against pathogens and damage (Buchon et al., 2009, 2013; Marianes and Spradling, 2013). ISCs support midgut intestinal cellular homeostasis by dividing throughout the entire lifespan of a fly when there is need for renewal, typically producing one renewed ISC and one enteroblast daughter cell. The enteroblast can subsequently differentiate into either an enterocyte or an enteroendocrine cell; the decision towards the two distinctive cell fates is determined by differential Notch pathway activation in the enteroblast (Ohlstein and Spradling, 2007; Perdigoto et al., 2011). A low Notch signal emanating from enteroendocrine cell daughters is also required to maintain multipotency of ISCs (Guo and Ohlstein, 2015). Additionally, a number of signalling pathways promote or restrict ISC proliferation in the fly midgut, including the Janus kinase/signal transducer and activator of transcription (JAK/STAT), Hippo, Jun N-terminal kinase (JNK), Wingless (Wg), Epidermal growth factor receptor (EGFR) and Insulin receptor signalling pathways. These pathways regulate ISC proliferation, maintenance and differentiation to ensure gut repair and remodelling in response to different stresses, such as injury, environmental damage and infection (Amcheslavsky et al., 2009; Biteau et al., 2008; Jiang and Edgar, 2009; Jiang et al., 2009; Lin et al., 2008; Shaw et al., 2010). Enterocytes regulate intestinal regeneration following intestinal damage or injury. The production of unpaired cytokines by stressed or damaged enterocytes leads to activation of the JAK/STAT pathway in ISCs, representing one example of how enterocytes non-autonomously influence ISC cell proliferation and renewal of the gut epithelium (Buchon et al., 2010; Jiang et al., 2009).

The role of ISCs in maintaining homeostasis under conditions of stress, damage or bacterial infection has been well studied. Interestingly, reduced nutrient availability decreases the abundance of intestinal enterocytes, slows down ISC cell division rate and consequently influences the size and length of the entire organ (O'Brien et al., 2011). Moreover, a protein poor diet results in greatly reduced enterocyte endoreplication, demonstrating that dietary protein is required for enterocyte turnover and/or differentiation (Britton and Edgar, 1998). Finally, modulation of enterocyte growth via insulin signalling can cell non-autonomously regulate ISC proliferation (Choi et al., 2011). These studies suggested that the growth status of enterocytes may influence ISC behaviour and midgut homeostasis. To further investigate this link we utilised the growth regulating properties of the *Drosophila* CyclinD/Cdk4 complex and of the mTOR-signalling pathway in order to genetically investigate the effects of enterocyte growth repression or activation on midgut homeostasis. CyclinD (CycD) and its kinase partner Cyclin dependent kinase 4 (Cdk4) control body size of adult flies and adult organs via control of cellular growth (accumulation of mass) in post-mitotic tissues (Emmerich et al., 2004; Meyer et al., 2000). Ectopic expression of CycD/Cdk4 increases the ploidy of highly endoreplicative tissues such as the larval salivary gland and the fat body (Datar et al., 2000; Frei et al., 2005). However, the consequence of CycD/Cdk4-driven growth varies depending on the cell type, causing accelerated cell division without affecting cell size in undifferentiated proliferating wing imaginal cells, while increasing cell size of post mitotic cells in differentiated eyes (Datar et al., 2000). Despite influencing cell cycle phasing when over-expressed, the *Drosophila* CycD/Cdk4 complex is dispensable for cell cycle progression (Meyer et al., 2000). While it is known that the kinase activity of Cdk4 is necessary for the growth promoting effects of CycD/Cdk4 (Meyer et al., 2000), the precise substrates that mediate these activities remain unclear. Growth promotion is however known to be genetically-independent of Rbf1 (Meyer et al., 2000) but dependent on mitochondrial ribosomal protein mRpL12 (Frei et al., 2005) and on Hif prolyl hydroxylase (Hph) (Frei and Edgar, 2004).

Here we show that intestinal stem cell proliferation and the concomitant cellular differentiation of daughter cells are partly dependent on cell autonomous Cdk4 functions but can also be regulated cell non-autonomously via control of enterocyte growth by the *Drosophila* CycD/Cdk4 complex and by manipulation of mTOR-dependent cellular growth.

## RESULTS

### Loss of Cdk4 causes loss of differentiated cells and impairs enterocyte endoreplication

**Fig. 1.**
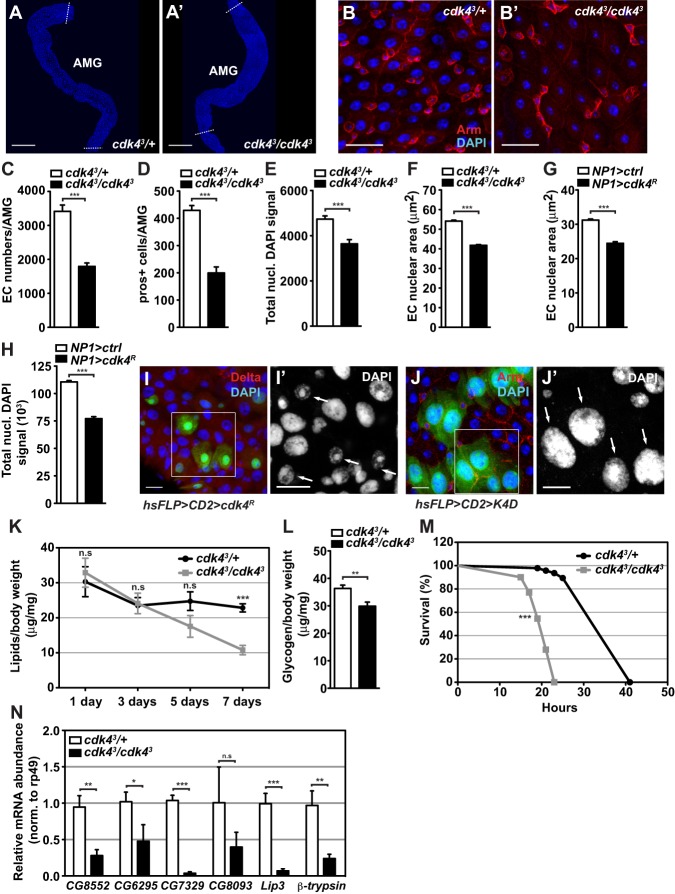
**Loss of Cdk4 in the adult midgut causes loss of differentiated cells and growth defective enterocytes.** (A,A′) DAPI-stained whole anterior midguts (AMG) from control and *cdk4^3^* mutant animals. Dotted lines indicate the beginning and end of AMG (middle midgut and posterior midgut not shown). Scale bars: 25 µm. (B,B′) Immunofluorescence staining of AMGs from mature adults (7-8 days old) using anti-Armadillo (Arm) and DAPI. Scale bars: 10 µm. (C-F) Quantifications of (C) numbers of polyploid enterocyte (EC) nuclei, (D) numbers of prospero-positive (pros+) enteroendocrine cells, (E) nuclear DAPI fluorescence signal (average per nucleus) and (F) nuclear area of enterocytes in the AMG. Genotypes in G and H: *NP1*-*Gal4/+* (*NP1>ctrl*), *NP1*-*Gal4/UAS-Cdk4^dsRNAi^* (*NP1>cdk4^R^*). (G) Nuclear DAPI fluorescence signal of enterocytes and (H) enterocyte nuclear area in AMGs with *NP1-Gal4-*driven *Cdk4* RNAi. (I-J) Flip-out clones expressing *Cdk4*-RNAi (I) or co-overexpressing CycD and Cdk4 (J) in 8-day-old adult flies stained with DAPI and anti-Delta (I) or with DAPI and anti-Armadillo (J). Arrows show individual clones, I′ and J′ are zooms of the marked boxes. Genotypes: *hsFLP,UAS-GFP;Act>CD2>Gal4,UAS-GFP; UAS-Cdk4^dsRNAi^* (*hsFLP>CD2>cdk4^R^*) and *hsFLP,UAS-GFP;Act>CD2>Gal4, UAS-GFP;UAS-CycD,UAS-Cdk4 (hsFLP>CD2>K4D)*. Scale bars: 10 µm. (K) Total body lipids of whole flies normalised against body weight. (L) Total body glycogen normalised against body weight in 7-8-day old adults. (M) Percentage survival of mature adult flies over time after complete food withdrawal. (N) Gene expression of lipases and *β-trypsin* quantified by qRT-PCR and measured in midguts of adult flies. *Lipase 3* (*Lip3*). Mean+s.d. shown in C,D,K,L,N; mean+s.e.m. shown in E-H; mean shown in M. *P<0.05, **P<0.01 and ***P<0.001; n.s., not significant.

To investigate the consequences of loss of CycD/Cdk4 functions on gut homeostasis we employed a null allele of *Cdk4* (*cdk4^3^*) and examined the guts of mature (7-day old) adult mutant males and age-matched controls. *cdk4^3^* homozygous mutant midguts displayed shortened anterior midguts (AMG, located after the foregut and prior to the middle midgut region) (Fig. 1A,A′) with an epithelium that is sparsely populated with cells compared to *cdk4^3^* heterozygous control guts (Fig. 1B,B′). We focused our further studies on the three sub regions of the AMG as this section contains the largest and most polyploid enterocytes in the gut and since *cdk4^3^* midguts show shortening of this region. *cdk4^3^* AMGs contain half the number of enterocytes (Fig. 1C) and enteroendocrine cells (Fig. 1D) of control guts. The number of enterocytes and enteroendocrine cells was also decreased in the posterior midgut region (data not shown). Since previous studies showed that *cdk4^3^* mutant animals have 10-20% less mass than heterozygous controls, and eyes and wings contain 10-15% fewer cells than controls (Datar et al., 2000; Meyer et al., 2000), we conclude that the AMG is disproportionately affected by loss of Cdk4 function. Importantly, adults mutant for *Cycd* (*cycd^1^*) showed very similar intestinal phenotypes to *cdk4^3^* mutants in terms of dramatically reduced abundance of differentiated cells (Fig. S1A-C). This argues that the observed *cdk4^3^* phenotypes are CycD-dependent functions of Cdk4, in agreement with previous studies that concluded that *Drosophila* CycD does not have any Cdk4-independent functions (Emmerich et al., 2004).

Enterocytes in the AMG region in *cdk4^3^* guts displayed significantly reduced endoreplication, as shown by decreased total DAPI fluorescence (Fig. 1E) and reduction in enterocyte nuclear area (Fig. 1F), suggesting that Cdk4 is required for endoreplication and normal enterocyte growth. To investigate if this requirement is cell autonomous we expressed RNAi against *Cdk4* (*cdk4^R^*) specifically in the enterocytes using the *Gal4* driver *NP1* (also known as *Myo1A-Gal4*) (Morgan et al., 1994). Indeed, a similar reduction in enterocyte nuclear area (Fig. 1G) and DAPI intensity (Fig. 1H) was observed in knockdown (*NP1>cdk4^R^*) intestines. We conducted clonal analysis experiments where *Cdk4* RNAi (*hsFLP>CD2>cdk4^R^*) or CycD and Cdk4 co-overexpression (*hsFLP>CD2>K4D*) were spontaneously induced in random clones using the hsFLP>Act>CD2 system. GFP marked clones expressing RNAi against *Cdk4* showed repressed endoreplication (Fig. 1I), while individual GFP clones overexpressing CycD/Cdk4 displayed increased enterocyte endoreplication without affecting the surrounding cells (Fig. 1J). We conclude that Cdk4 is required cell autonomously for enterocyte endoreplication and growth.

In addition to the observed midgut cellular phenotypes, *cdk4^3^* adults show signs of compromised intestinal health and function. This is manifested through reduced nutritional storage, measured as total body lipids (Fig. 1K) and glycogen (Fig. 1L). The reduction in stored nutrients renders *cdk4^3^* adults highly starvation sensitive upon complete food deprivation (Fig. 1M). Furthermore, *cdk4^3^* midguts show significantly reduced gene expression of several midgut lipases (Fig. 1N), which are enzymes that hydrolyse dietary and stored lipids (Asher et al., 1984; Grönke et al., 2005, 2007). General functional deterioration of the fly intestinal mucosa can be assessed by analysing expression levels of several trypsin genes expressed in the gut. The expression of the most abundant trypsin gene, β-trypsin, was reduced by 75% in mutant guts compared to controls (Fig. 1N). This is comparable to the decrease in expression seen in 30-day-old fly intestines compared to 3-day-old intestines, where the reduction was around 60% and was linked to age-dependent decline in gut function (Biteau et al., 2008).

In summary, we show that loss of function of the *Drosophila* CycD/Cdk4 complex in the adult midgut causes substantial loss of differentiated cells and that this complex additionally controls enterocyte cellular growth and endoreplication entirely cell autonomously.

### Cdk4 expression is required for efficient ISC proliferation and differentiation

**Fig. 2.**
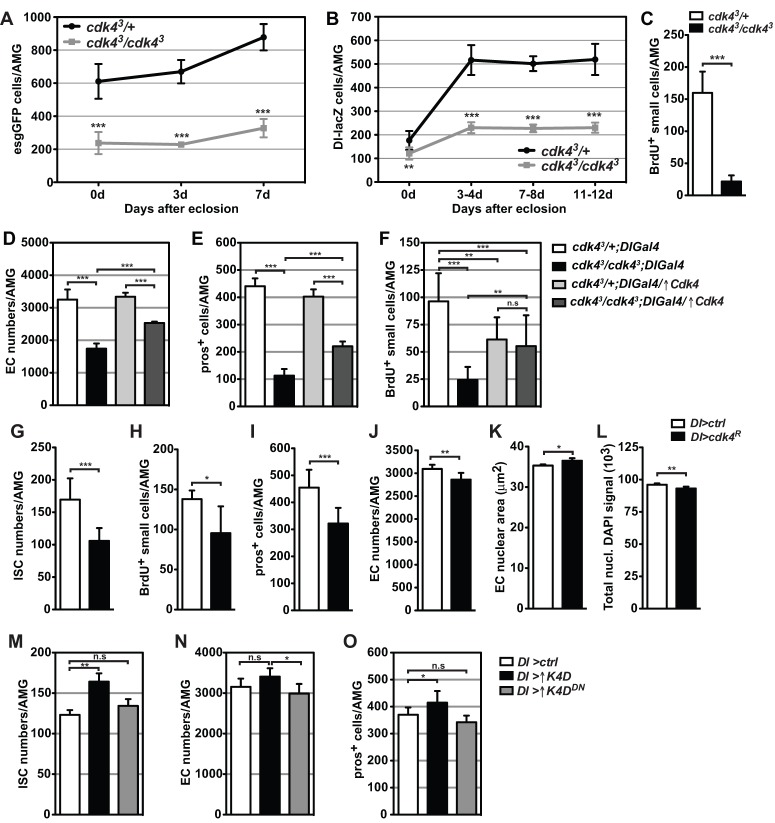
**ISCs require Cdk4 expression for correct proliferation and differentiation.** (A-C) Numbers of (A) esgGFP-positive cells, (B) Dl-lacZ-positive cells and (C) BrdU+ small cells in AMGs of *cdk4^3^* mutants and controls. Genotypes in A: *esgGFP-Gal4-cdk4^3^/+* and *esgGFP-Gal4-cdk4^3^/cdk4^3^*. Genotypes in B: *cdk4^3^/+;Dl-lacZ* and *cdk4^3^/cdk4^3^;Dl-lacZ*. (D-F) Numbers of (D) enterocytes (EC), (E) pros+ enteroendocrine cells, (F) BrdU+ small cells in AMGs expressing a *Dl-Gal4*-driven *Cdk4* transgene in *cdk4^3^* mutants and controls. ↑Cdk4 refers to over-expression of Cdk4. (G-L) Numbers of (G) ISCs, (H) BrdU+ small cells, (I) pros+ enteroendocrine cells, (J) enterocytes, (K) enterocyte nuclear area and (L) enterocyte DAPI fluorescent signal in AMGs with Cdk4 knockdown in ISCs using the *Delta-Gal4* driver (*Dl-Gal4*). Genotypes: *Dl-Gal4/+* (*Dl>ctrl*) and *Dl-Gal4/UAS-Cdk4^dsRNAI^* (*Dl>cdk4^R^*). (M-O) Numbers of (M) ISCs, (N) enterocytes and (O) pros+ enteroendocrine cells in AMGs with overexpression of CycD/Cdk4 (*Dl>K4D*) or CycD/Cdk4 kinase dead dominant negative (*Dl>K4D^DN^*). ISC numbers in G and M were derived from driving *UAS-GFP* with *Delta-Gal4*. Mean+s.d.; *P<0.05, **P<0.01 and ***P<0.001; n.s., not significant.

Given the severe deficiency in differentiated cells in *cdk4^3^* mutant intestines, we examined the abundance of ISCs and enteroblasts by marking these cell populations with GFP using the driver *escargot*-*Gal4* (*esgGFP-Gal4*), which is strongly expressed in ISCs and enteroblasts (Micchelli and Perrimon, 2006) but also very weakly in immature enterocytes in adult midguts (Shaw et al., 2010). *cdk4^3^* mutant midguts displayed a significant reduction in the number of GFP positive cells in both immature and mature intestines compared to controls (approximately 60% fewer cells in the AMG), suggesting that the entire ISC and enteroblast populations are greatly reduced (Fig. 2A). In order to directly quantify the number of ISCs, Delta (Dl)-lacZ was expressed in control and *cdk4^3^* mutant backgrounds (Beebe et al., 2010; Zeng et al., 2010). Quantification of lacZ+ cells revealed significantly decreased numbers of ISCs in *cdk4^3^* AMGs at all examined ages (Fig. 2B). The difference in ISC numbers between mutants and controls was less prominent in guts of newly eclosed adults, however, while ISC numbers dramatically increased between 0 and 3-4 days of age in wild type AMGs, the numbers of ISCs in *cdk4^3^* mutant AMGs increased only moderately, resulting in a 56% reduction in ISC numbers in 7-day-old intestines. This implied a possible impairment of ISC proliferation and self-renewal. To test this idea, 2-day-old flies were fed 5-bromo-2-deoxyuridine (BrdU) for 48 h and progenitor cells were identified based on their characteristic small nuclear size. Counting of small BrdU labelled cells demonstrated that 85% fewer ISCs and enteroblasts incorporated BrdU in *cdk4^3^* midguts compared to controls, revealing an abnormally low proliferative rate under conditions of normal gut homeostasis (Fig. 2C). The reduced ISC self-renewal is not exclusively an age-dependent phenotype as it was also observed when feeding was initiated with older *cdk4^3^* adults (Fig. S2A). The proliferative capacity of *cdk4^3^* ISCs was further investigated through artificial damage induction by feeding adult flies with dextran sodium sulfate (DSS). DSS feeding in flies leads to increased ISC cell division and marked increase in enteroblast numbers (Amcheslavsky et al., 2009). *cdk4^3^* mutant guts expressing *esgGFP-Gal4* responded to DSS feeding by strongly increasing the abundance of progenitor cells, which accumulate in numerous esgGFP*+* cell clusters (Fig. S2B), demonstrating that mutant ISCs are capable of responding to damage-induced proliferation cues, in contrast to the suppressed self-renewal rate observed under normal conditions.

To further clarify the cellular specificity of Cdk4 function in intestinal homeostasis we genetically manipulated Cdk4 or CycD/Cdk4 expression in various midgut cell types in *cdk4^3^* mutant and control backgrounds using the UAS-Gal4 system. Cdk4 re-expression in ISCs of *cdk4^3^* flies using the *Dl*-*Gal4* driver (*cdk4^3^*/*cdk4^3^*;*DlGal4;*↑*Cdk4*) resulted in significantly increased, yet not completely rescued enterocyte (Fig. 2D) and enteroendocrine cell (Fig. 2E) cell numbers when compared to *cdk4^3^* midguts without transgene expression (*cdk4^3^*/*cdk4^3^*;*DlGal4*). BrdU feeding experiments demonstrated a doubling of the number of proliferating progenitor cells in the *Cdk4* rescued *cdk4^3^* midguts compared to *cdk4^3^* mutant controls (Fig. 2F). Total ISC numbers could not be quantified in this experimental setting due to combinatorial lethality of the insertions in the *Dl-lacZ* and the *Dl-Gal4* alleles. Complementary RNAi experiments were conducted with *Cdk4* knock-down specifically in ISCs using *Dl-Gal4* (*DI>cdk4^R^*), resulting in a reduction in ISC numbers (Fig. 2G) and concomitant repression of ISC self-renewal as shown by BrdU labelling (Fig. 2H). These AMGs displayed a 30% reduction in enteroendocrine cell numbers (Fig. 2I) and a 7.5% reduction in enterocyte numbers (Fig. 2J). Enterocyte nuclear area (Fig. 2K) and endoreplication (Fig. 2L) displayed very minor alterations. ISC-specific overexpression of CycD/Cdk4 in wild type flies induced a 15% increase in ISC numbers (Fig. 2M), a non-significant trend towards increased enterocyte numbers (Fig. 2N) and an increase in enteroendocrine cell numbers (Fig. 2O), consistent with a pro-proliferative role of Cdk4 in progenitor cells. These effects were not observed when CycD was co-overexpressed with a kinase dead Cdk4 mutant (Fig. 2M-O).

Altogether, the ISC rescue, knock-down and overexpression experiments demonstrate that there is a cell autonomous requirement for Cdk4 activity in ISCs for correct regulation of ISC proliferation and generation of enteroendocrine cells and enterocytes, but also show that the complete null phenotype involves additional effects of Cdk4 activity in other cell types.

**Fig. 3.**
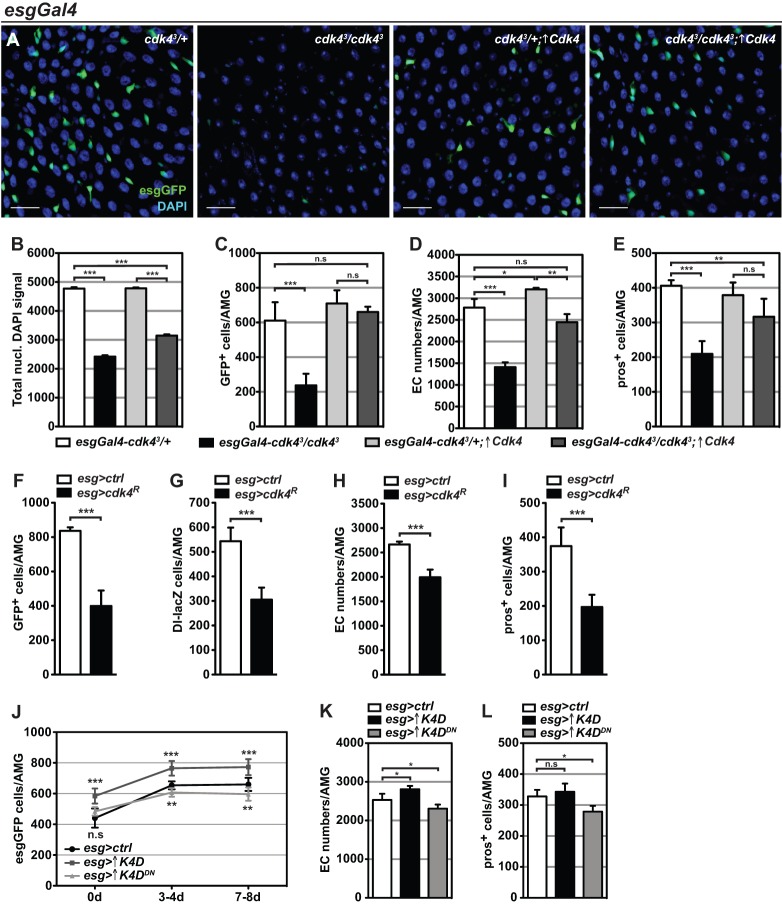
**Cell-autonomous requirements of Cdk4 in ISCs/enteroblasts for regulation of proliferation and differentiation.** (A) DAPI and GFP fluorescence (marking ISCs and enteroblasts) of AMGs expressing a Cdk4 transgene driven by *esgGFP-Gal4* (*esgGal4*) in *cdk4^3^* mutants and controls. Scale bars: 10 µm. ↑Cdk4 refers to over-expression of Cdk4. (B-E) Quantifications of *esgGal4* rescue AMGs with respect to (B) enterocyte endoreplication, (C) ISC/enteroblast numbers, (D) enterocyte (EC) numbers and (E) enteroendocrine cell numbers. (F-I) Effect of *Cdk4* knockdown using *esg-Gal4* on numbers of (F) ISC/enteroblasts, (G) ISCs, (H) enterocytes and (I) enteroendocrine cells. Genotypes: *esgGFP-Gal4/+* (*esg>ctrl*) and *esgGFP-Gal4/UAS-Cdk4^dsRNAi^* (*esg>cdk4^R^*). (J-L) Numbers of (J) ISCs/enteroblasts, (K) enterocytes and (L) pros+ enteroendocrine cells in AMGs with overexpression of CycD/Cdk4 (*esg>*↑*K4D*) or CycD/Cdk4 kinase dead dominant negative (*esg>*↑*K4D^DN^*) using the *esg-GFP-Gal4* driver. Genotypes in J-L: *esgGFP-Gal4/+* (*esg>ctrl*), *esgGFP-Gal4;UAS-CycD*, *UAS-Cdk4* (*esg>↑K4D*) and *esgGFP-Gal4;UAS-CycD*, *UAS-Cdk4^D175N^* (*esg>↑K4D^DN^*). Mean+s.d.; *P<0.05, **P<0.01 and ***P<0.001; n.s., not significant.

To evaluate the collaborative function of Cdk4 in ISCs and enteroblasts, Cdk4 was re-expressed using the *esgGFP-Gal4* driver in *cdk4^3^* intestines (*esgGal4-cdk4^3^*/*cdk4^3^;*↑*Cdk4*). This resulted in an apparent rescue of the epithelial appearance compared to *cdk4^3^* mutants (*esgGal4-cdk4^3^*/*cdk4^3^*) with respect to enterocyte nuclear morphology and occurrence of GFP positive progenitor cells (Fig. 3A). Quantitative analysis revealed that these midguts indeed show significantly increased enterocyte nuclear area (Fig. S2C) compared to control *cdk4^3^* midguts and a partial rescue of endoreplication (Fig. 3B). With respect to cellular abundance, the number of ISCs/enteroblasts was entirely rescued (Fig. 3C) and the number of differentiated enterocytes (Fig. 3D) and enteroendocrine cells (Fig. 3E) was not entirely, but significantly restored. Furthermore, induction of Cdk4 RNAi using *esgGFP-Gal4* (*esg>cdk4^R^*) could mimic multiple *cdk4^3^* midgut phenotypes with respect to significantly decreased numbers of GFP+ cells (ISCs/enteroblasts) (Fig. 3F), as well as specifically reduced numbers of DI-lacZ+ ISCs (Fig. 3G) and differentiated enterocytes (Fig. 3H) and enteroendocrine cells (Fig. 3I). Overexpression of CycD/Cdk4 using *esgGFP-Gal4* in wild type guts moderately increased the number of progenitor cells marked by esg-GFP (Fig. 3J) and increased the number of enterocytes (Fig. 3K) but not enteroendocrine cells (Fig. 3L). Overexpression of CycD together with kinase dead Cdk4, which can act in some settings as a dominant negative (Meyer et al., 2000), caused a slight reduction in progenitor, enterocyte and enteroendocrine cell numbers (Fig. 3J-L), consistent with the results obtained using *Cdk4* RNAi. These results collectively demonstrate the cell autonomous proliferation-promoting activities of CycD/Cdk4 in progenitor cells.

Enteroblast-specific repression of *Cdk4* using the *Suppressor of hairless* (*Su(H)Gbe*)-*Gal4* driver did not affect gut enterocyte numbers (Fig. S2D), however it significantly decreased the number of enteroendocrine cells (Fig. S2E), suggesting that Cdk4 function in enteroblasts is necessary to ensure efficient formation of enteroendocrine cells.

While these findings argue that the combined function of Cdk4 in ISCs and enteroblasts is important for maintaining intestinal epithelial homeostasis, a potential caveat is that the *esgGFP-Gal4* driver also induces expression in early enterocytes. Since the *esgGFP-Gal4* Cdk4 rescue experiment shows partially restored enterocyte endoreplication and growth, possibly due to persistence of transgene expression in early enterocytes, the apparent rescue of ISC and enteroblast numbers and partial rescue of differentiation could also potentially be a secondary consequence of inducing enterocyte growth.

### Enterocyte growth regulates ISC proliferation and differentiation

**Fig. 4.**
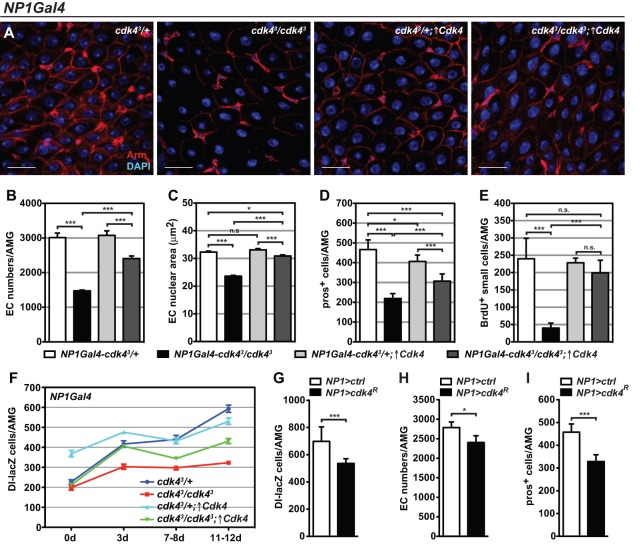
**Cdk4 cell-autonomously controls enterocyte growth and endoreplication and cell-non-autonomously controls ISC self-renewal.** (A) Immunofluorescence staining of AMGs expressing Cdk4 using *NP1-Gal4* in *cdk4^3^* mutants and controls. Arm, anti-Armadillo. Scale bars: 20 µm. (B-F) Quantifications of (B) enterocyte (EC) numbers, (C) enterocyte nuclear area, (D) pros+ enteroendocrine cells, (E) BrdU+ small cells and (F) Dl-lacZ+ ISCs in AMGs expressing Cdk4 using *NP1-Gal4* in *cdk4^3^* mutants and controls. Genotypes in F: *NP1-Gal4-cdk4^3^/+;Dl-lacZ* (*cdk4^3^/+*), *NP1-Gal4-cdk4^3^/cdk4^3^;Dl-lacZ* (*cdk4^3^/cdk4^3^*), *NP1-Gal4-cdk4^3^/+;UAS-Cdk4/Dl-lacZ* (*cdk4^3^/+;*↑*Cdk4*), *NP1-Gal4-cdk4^3^/cdk4^3^;UAS-Cdk4/Dl-lacZ* (*cdk4^3^/cdk4^3^;↑Cdk4*). ↑Cdk4 refers to over-expression of Cdk4. (G-I) Numbers of (G) Dl-lacZ+ ISCs, (H) enterocytes and (I) pros+ enteroendocrine cells in AMGs with *NP1-Gal4*-driven *Cdk4* knockdown. Genotypes in H and I: *NP1*-*Gal4*/+ (*NP1>ctrl*), *NP1*-*Gal4/UAS-Cdk4^dsRNAi^* (*NP1>cdk4^R^*). Genotypes in G: *NP1-Gal4/+;Dl-lacZ* (*NP1>ctrl*) and *NP1-Gal4/UAS-Cdk4^dsRNAi^;Dl-lacZ*. Mean+s.d. shown in B,D-I; mean+s.e.m. shown in C; *P<0.05, **P<0.01 and ***P<0.001; n.s., not significant.

To address the specific role of Cdk4 in enterocyte growth control and the consequent effects on gut homeostasis, Cdk4 was expressed using the enterocyte-specific *NP1-Gal4* driver in *cdk4^3^* mutant (*NP1Gal4-cdk4^3^*/*cdk4^3^;* ↑*Cdk4*) or control backgrounds (*NP1Gal4-cdk4^3^*/*+;* ↑*Cdk4*). In the mutant background, the abnormal sparse cellular appearance of the *cdk4^3^* epithelium was rescued (Fig. 4A). There was a significant increase in enterocyte numbers (Fig. 4B) and enterocyte nuclear area (Fig. 4C) and endoreplication defects were rescued (Fig. S3A). Enteroendocrine cell numbers were only partially restored (Fig. 4D). The proliferative rate of ISCs increased drastically and showed a similar extent of BrdU incorporation to control midguts (Fig. 4E). Additionally, the abundance of ISCs was entirely rescued in immature and very young guts and partially but significantly restored in mature guts (Fig. 4F). Complementing these findings, RNAi-mediated knockdown of *Cdk4* in the enterocytes (*NP1>cdk4^R^*) significantly decreased the abundance of ISCs in the AMG region (Fig. 4G). These midguts also displayed a significant reduction in differentiated enterocytes (Fig. 4H) and enteroendocrine cells (Fig. 4I), as well as a non-significant but noteworthy tendency towards less ISC proliferation (Fig. S3B). Moreover, mutant adults with enterocyte-specific Cdk4 re-expression showed a complete rescue of lipid levels (Fig. S3C), suggesting that *cdk4^3^* metabolic phenotypes are specifically due to loss of Cdk4 in enterocytes.

In summary, the role of Cdk4 in enterocytes is not exclusively restricted to cell-autonomously controlling enterocyte endoreplication and growth but it also influences ISC proliferation and differentiation cell non-autonomously.

**Fig. 5.**
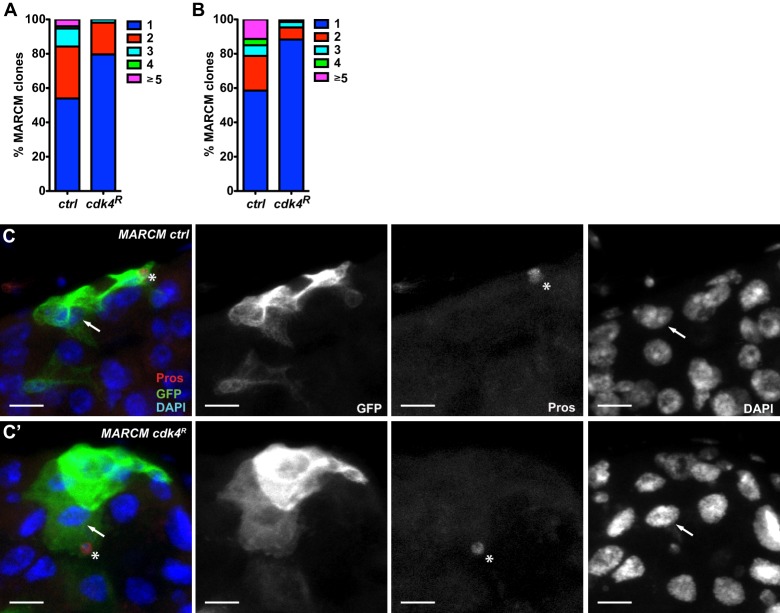
***Cdk4*** **RNAi MARCM stem cell clones fail to expand.** (A-C) Distributions of numbers of cells per clone of MARCM control and RNAi clones at (A) 3 days (76 control clones in eight guts and 54 RNAi clones in seven guts) and (B) 32 days (405 control clones in 11 guts and 385 RNAi clones in 12 guts) after clone induction. (C) Examples of GFP-marked clones (green) containing pros+ enteroendocrine cells (red, marked with *) and polyploid enterocytes (marked with arrow). Genotypes: *hsFLP, UAS-GFP;+;tubGal4, FRT82, tubGal80/FRT82* (*MARCM ctrl*) and *hsFLP, UAS-GFP;UAS-Cdk4^dsRNAi^;tubGal4, FRT82, tubGal80/FRT82* (*MARCM cdk4^R^*). Scale bars: 10 µm.

To further characterise the functions of Cdk4 in the gut we conducted ISC clonal analyses. It was unfortunately not possible to induce MARCM stem cell clones in the *cdk4^3^* mutant background because of the frequent lethality of *cdk4^3^* homozygous adults when subjected to heat-shock and because clone induction in the few surviving animals was virtually zero as a result of the extremely low rates of ISC proliferation in the mutant background. We therefore induced MARCM stem cell clones expressing *Cdk4* RNAi to analyse the effect of loss of Cdk4 function in individual ISCs and their differentiated derivatives in the context of surrounding wild type cells. *Cdk4* RNAi clones showed more single cell clones and fewer multicellular clones than control clones at 3 days and 32 days after clone induction (Fig. 5A,B), indicating defects in clonal expansion. In the few expanded *Cdk4* RNAi clones it was possible to identify enteroendocrine cells and enterocytes (Fig. 5C), indicating that the loss of Cdk4 function does not absolutely block differentiation. The rarity of these expanded clones prevented a quantitative assessment of the frequency of differentiation towards the enteroendocrine cell and enterocyte lineages. These results are consistent with the demonstrated cell autonomous contribution of Cdk4 to ISC proliferation and differentiation. Additionally, in contrast to the experiments showing that enterocyte-specific Cdk4 reintroduction into the *cdk4^3^* mutant gut largely restores ISC proliferation, abundance and differentiation, in the clonal setting the presence of surrounding wild type enterocytes does not support the efficient clonal expansion of *Cdk4* RNAi ISCs, arguing that Cdk4 function is specifically necessary in the daughter enteroblasts and/or enterocytes to maintain correct ISC function.

**Fig. 6.**
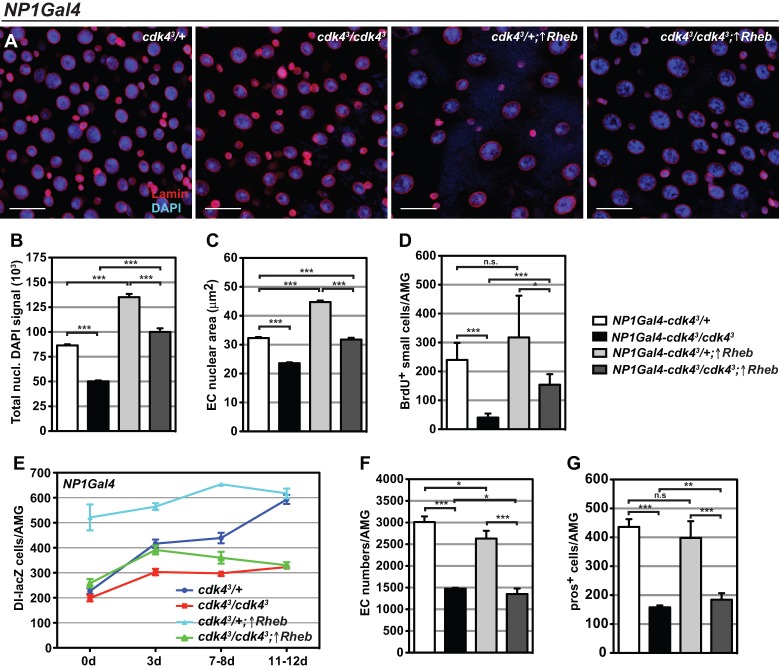
**Ectopic expression of Rheb drives enterocyte growth in *cdk4^3^* enterocytes but does not rescue mis-differentiation of *cdk4^3^* mutant ISCs.** AMGs of flies over-expressing Rheb using *NP1-Gal4* in *cdk4^3^* mutants and controls were (A) stained DAPI and anti-Lamin (scale bars: 20 µm) and quantified for (B) nuclear DAPI fluorescence signal of enterocytes (EC), (C) enterocyte nuclear area, (D) numbers of BrdU+ small cells, (E) numbers Dl-lacZ positive ISCs, (F) numbers of enterocytes and (G) numbers of pros+ enteroendocrine cells. Genotypes: *NP1-Gal4-cdk4^3^/+;Dl-lacZ* (*cdk4^3^/+*), *NP1-Gal4-cdk4^3^/cdk4^3^;Dl-lacZ* (*cdk4^3^/cdk4^3^*), *NP1-Gal4-cdk4^3^/+;Dl-lacZ/UAS-Rheb* (*cdk4^3^/+;*↑*Rheb*), *NP1-Gal4-cdk4^3^/cdk4^3^;Dl-lacZ/UAS-Rheb* (*cdk4^3^/cdk4^3^;*↑*Rheb*). ↑Rheb refers to over-expression of Rheb. Mean+s.d. shown in B-D,F,G; mean+s.e.m. shown in E; *P<0.05, **P<0.01 and ***P<0.001; n.s., not significant.

To address if loss of proliferation and proper differentiation in the *cdk4^3^* epithelium is strictly due to absence of Cdk4-dependent cues from the enterocytes or a consequence of general enterocyte growth impairment, enterocyte growth was induced via ectopic over-expression of Rheb. *Drosophila* Rheb (Ras homolog enriched in brain) induces growth in multiple tissues when over-expressed in the developing fly (Stocker et al., 2003). Visual inspection revealed that Rheb-expressing *cdk4^3^* mutant epithelium showed no noticeable change in appearance compared to mutant epithelium without Rheb overexpression with respect to the sparse cellular appearance of the epithelium in 7-8-day-old midguts (Fig. 6A). However, induction of Rheb expression in enterocytes drives endoreplication in both the control (*NP1Gal4-cdk4^3^/+*;↑*Rheb*) and *cdk4^3^* mutant (*NP1Gal4-cdk4^3^/cdk4^3^*;↑*Rheb*) backgrounds, as shown by increased DAPI intensity (Fig. 6B) and nuclear area (Fig. 6C). Consistent with our data showing that enterocyte growth status regulates ISC proliferation and abundance, Rheb overexpression in enterocytes of control flies showed a non-significant tendency to increase the number of proliferating small cells (Fig. 6D) and dramatically increased the total number of ISCs per midgut (Fig. 6E). The enterocyte-specific expression of Rheb in *cdk4^3^* mutant midguts had a similar effect and significantly increased the number of small BrdU+ cells (Fig. 6D) and increased the number of Dl-lacZ*+* cells in immature 3-day-old midguts compared to *cdk4^3^* controls at the same age (Fig. 6E). Interestingly, this initial strong expansion of the midgut stem cell pool in the Rheb-expressing flies is gradually reversed as animals mature and eventually declines to the same ISC numbers as seen in *cdk4^3^* intestines by about 12 days of age. Quantifications of total differentiated cells in these midguts confirmed there was no increase in the numbers of enterocytes in experimental mutants compared to *cdk4^3^* mutant controls without Rheb overexpression (Fig. 6F) and enteroendocrine cell abundance was only very marginally increased (Fig. 6G).

Thus, *cdk4^3^* ISCs surrounded by Rheb-overexpressing, growth-rescued mutant enterocytes are induced to initially proliferate but neither seem to be maintained, nor do they end up as differentiated cells. ISC proliferation and differentiation appears to be governed partly by the growth-status of enterocytes but also partly by other Cdk4-dependent functions or signals from enterocytes.

**Fig. 7.**
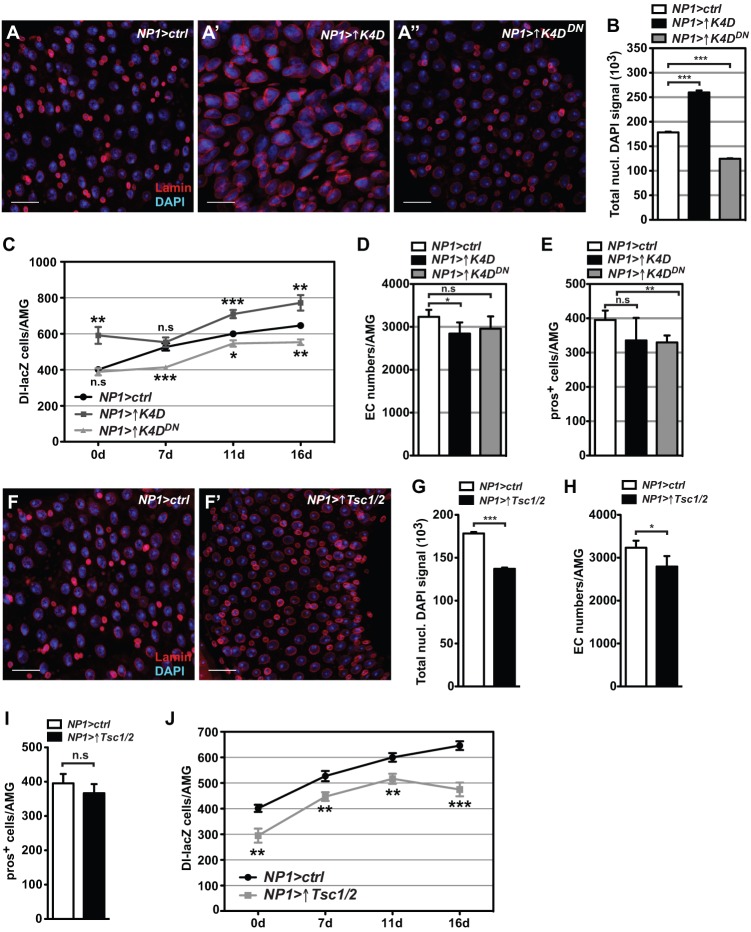
**Enterocyte growth modulation by CycD/Cdk4 and Tsc1/Tsc2 over-expression cell-non-autonomously influences ISC abundance and differentiation.** (A-E) AMGs co-overexpressing transgenes of either CycD/Cdk4 (A′) or CycD with a kinase inactive Cdk4 (A″) using *NP1-Gal4* were stained for DAPI and anti-Lamin and quantified for (B) nuclear DAPI fluorescence signal of enterocytes, (C) numbers of Dl-lacZ+ cells, (D) numbers of enterocytes (EC) and (E) numbers of pros+ enteroendocrine cells per AMG. (F-J) AMGs co-over-expressing a Tsc1/Tsc2 transgene using *NP1-Gal4* were stained for (F) DAPI and anti-Lamin and quantified for (G) nuclear DAPI fluorescence signal of enterocytes, (H) numbers of enterocytes, (I) numbers of pros+ enteroendocrine cells and (J) numbers of DI-lacZ+ cells per AMG. Genotypes in A,B, D,E: *NP1-Gal4/+* (*NP1>ctrl*), *NP1-Gal4/UAS-CycD,UAS-Cdk4* (*NP1>*↑*K4D*), *NP1-Gal4/UAS-CycD,UAS-Cdk4^D175N^* (*NP1>*↑*K4D^DN^*). Genotypes in F-I: *NP1-Gal4/+* (*NP1>ctrl*) and *NP1-Gal4/UAS-Tsc1*, *UAS-Tsc2* (*NP1>*↑*Tsc1/2*). Genotypes in C,J additionally include Delta-lacZ (Dl-lacZ). ↑Tsc1/2, ↑K4D and ↑K4D^DN^ refers to over-expression of these transgenes. Scale bars: 20 µm.Mean+s.d. shown in C-E,H-J; mean+s.e.m. shown in B,G; *P<0.05, **P<0.01 and ***P<0.001; n.s., not significant.

In order to further investigate general effects of changes in enterocyte growth on the proliferation and differentiation of wild type ISCs, enterocyte growth was activated by ectopic expression of the CycD/Cdk4 complex. CycD/Cdk4 co-overexpression can induce overgrowth phenotypes in postmitotic cells of the adult eye and endoreplicating cells in the larval salivary gland and the fat body (Datar et al., 2000; Frei et al., 2005). enterocyte specific co-overexpression of CycD/Cdk4 (*NP1>*↑*K4D*) caused overgrowth of enterocytes in the entire midgut compared to control midguts (Fig. 7A,B and Fig. S3D), whereas co-overexpression of CycD and a kinase-inactive Cdk4 (*NP1>*↑*K4D^DN^*) abolished the induction of nuclear growth (Fig. S3D) and caused decreased DAPI intensity (Fig. 7B), implying repression of endoreplication, consistent with a dominant negative function of kinase inactive Cdk4 (Meyer et al., 2000). Similar to the effect of driving enterocyte growth with Rheb, driving enterocyte growth by modifying levels of CycD/Cdk4 increases the overall number of ISCs in the AMG (Fig. 7C), while ectopic expression of the kinase-inactive Cdk4 causes a decrease in the number of ISCs (Fig. 7C), mimicking the effect of *Cdk4* RNAi. The abundance of differentiated enterocytes (Fig. 7D) and enteroendocrine cells (Fig. 7E) is marginally but significantly decreased in midguts either expressing the kinase active or inactive Cdk4. Flies with *NP1Gal4*-driven expression of CycD/Cdk4 displayed the opposite nutrient phenotype to *cdk4^3^* mutant flies, namely higher levels of stored glycogen (Fig. S3E) and lipids (Fig. S3F).

To complement these findings, we modulated enterocyte growth by genetically manipulating the mTOR-dependent growth pathway. The *Drosophila* Tuberous sclerosis complex 1 and 2 proteins (Tsc1/2) negatively regulate the Target of rapamycin (mTOR) signalling pathway downstream of Akt, but upstream of Rheb, S6K and 4E-BP (Napolioni and Curatolo, 2008). Ectopic co-expression of Tsc1 and Tsc2 using *NP1-Gal4* (*NP1>*↑*Tsc1/2*) repressed enterocyte growth, demonstrated by decreased nuclear area (Fig. 7F; Fig. S3G) and endoreplication (Fig. 7G). These midguts also display marginally but significantly reduced numbers of differentiated enterocytes (Fig. 7H) and a tendency towards fewer enteroendocrine cells (Fig. 7I). Importantly, the abundance of ISCs is reduced in immature (26.5%), mature (7-day-old, 15.2%) and older (16-17-day-old, 26.5%) Tsc1/2 AMGs compared to controls (Fig. 7J).

In summary, manipulating enterocyte growth status through CycD/Cdk4-dependent or -independent pathways exerts cell non-autonomous effects on the proliferation and abundance of wild type ISCs and smaller effects on their differentiation to form enterocytes and enteroendocrine cells. Major effects on the maintenance and/or differentiation of ISCs/enteroblasts to form enterocytes and enteroendocrine cells are seen when Cdk4 function is also deficient in ISCs/enteroblasts as well as in enterocytes.

### ISC differentiation defects are caused by Cdk4 deficiency

Since our experiments showing reduced overall enteroendocrine cell and enterocyte numbers suggest that ISCs residing in a *cdk4^3^* mutant gut epithelium exhibit multiple lineage differentiation defects, we investigated cell fate maintenance of ISCs and their daughter cells using a variety of molecular markers of stemness and cell lineage determination, including Delta transcriptional activity (Dl-lacZ reporter) and protein abundance, esgGFP presence and levels of Notch pathway activation (Su(H)Gbe-lacZ reporter). In mature *cdk4^3^* guts, across all AMG regions, we observed the occurrence of large esgGFP+ cells that simultaneously stain for Delta protein (Fig. S4A,A′). We performed a rescue experiment with enterocyte specific re-expression of Cdk4 and quantified the nuclear area of Dl-lacZ expressing ISCs. The area of lacZ+ nuclei demonstrated a shifted size distribution pattern towards larger nuclei in *cdk4^3^* mutant intestines compared to controls (Fig. S4B), as well as significantly increased mean nuclear area (Fig. S4C). This phenotype was entirely rescued in *cdk4^3^* mutant guts with enterocyte specific Cdk4 expression (Fig. S4B,C), demonstrating a cell non-autonomous effect of Cdk4 in enterocytes on the ISC cell compartment.

**Fig. 8.**
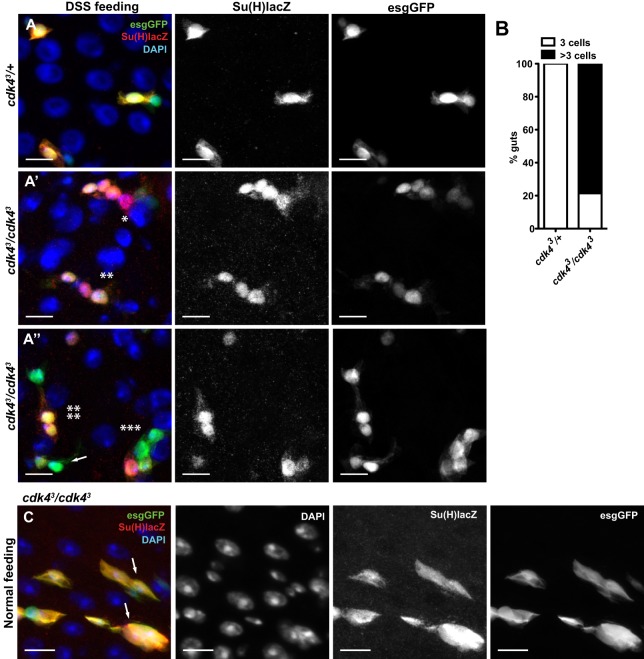
***cdk4^3^* mutant intestines accumulate cells with aberrant cell identity markers.** (A-A″) AMGs from DSS-fed flies stained for Notch reporter Su(H)Gbe-lacZ (Su(H)lacZ) (red) and esgGFP (green). In A′ and A″ asterisks denote chaining of cells and mis-expression of Su(H)Gbe-lacZ and esgGFP. In A″ single arrow indicates normal expression of markers with a Su(H)Gbe-lacZ*+* and esgGFP*+* enteroblast and a Su(H)Gbe-lacZ− and *esgGFP+* ISC. (B) Number of AMGs in which the maximum size of clusters of Su(H)Gbe-lacZ positive (marking ISCs and enteroblasts) small cells was three or more than three cells (*n*=12-14 guts). (C) AMGs from mutant flies fed on normal diet and stained against Su(H)Gbe-lacZ (red) and esgGFP (green). Arrows show enteroblast clusters. Genotypes in A-C: *Su(H)Gbe-lacZ;esgGFP-Gal4-cdk4^3^*/*+* (*cdk4^3^*/*+*) and *Su(H)Gbe-lacZ;esgGFP-Gal4-cdk4^3^*/*cdk4^3^* (*cdk4^3^*/*cdk4^3^*). Scale bars: 10 µm.

Given our observation that DSS feeding promotes the accumulation of clusters of small esgGFP+ cells in *cdk4^3^* mutant AMGs (Fig. S2B), we next sought to experimentally enhance ISC proliferation and thereby enhance the possibility of observing potential mis-differentiation phenotypes. This was achieved by feeding DSS to *cdk4^3^* mutant flies that also express both the Notch-responsive enteroblast marker Su(H)Gbe-lacZ and the ISC/enteroblast marker esgGFP. Su(H)Gbe-lacZ is commonly used in fly intestines to monitor the activation of the Notch pathway (Micchelli and Perrimon, 2006; Ohlstein and Spradling, 2006; Perdigoto et al., 2011). ISCs are normally esgGFP+ and Su(H)Gbe-lacZ− while enteroblasts are esgGFP+ and Su(H)Gbe-lacZ+. In *cdk4^3^* heterozygous damaged midguts, ISCs and enteroblasts were always observed in pairs or in clusters of maximally three Su(H)Gbe-lacZ+ cells (Fig. 8A). Clusters of more than three cells that were positive for Su(H)Gbe-lacZ were never observed (Fig. 8B). However, in the DSS-fed *cdk4^3^* mutant epithelium, in addition to normal ISC/enteroblast pairs (Fig. 8A″, arrow), cells expressing various combinations of esgGFP and Su(H)Gbe-lacZ were frequently observed in chains or clusters of more than 3 cells with small nuclei. 85% of *cdk4^3^* mutant guts displayed at least one such cell cluster in the AMG (Fig. 8B). Additionally, the combination of cell markers (esgGFP and Su(H)Gbe-lacZ) in these cell chains/clusters was aberrant in terms of the position of the cells in relation to one another and in terms of the combinations of expressed markers. For example, Fig. 8A′ (single asterisk) shows a chain of 6 small cells in which 4 of the cells display double labelling for Su(H)Gbe-lacZ and esgGFP, indicating enteroblasts, except for one cell that shows the abnormal pattern of Su(H)Gbe-lacZ+ and esgGFP− and one cell that appears to be an ISC [Su(H)Gbe-lacZ− and esgGFP+]. Other cases are found where multiple cells in a row contain adjacent cells that are doubly positive for esgGFP and Su(H)Gbe-lacZ, one small cell expresses Su(H)Gbe-lacZ but is very weakly positive for esgGFP and one small cell is negative for both markers (Fig. 8A′, double asterisk). Clusters of four cells with two apparent ISCs and two apparent enteroblasts (Fig. 8A″, triple asterisk), as well as examples of two adjacent enteroblasts without an adjacent ISC are found (Fig. 8A″, quadruple asterisk). These findings of chaining of progenitor cells and expression of aberrant patterns of lineage markers are consistent with a breakdown of the normal control of ISC and enteroblast differentiation in *cdk4^3^* mutant guts.

To investigate the contributions of Cdk4 in different cell types to these mis-differentiation phenotypes we induced *Cdk4* RNAi in ISCs (*Dl>cdk4^R^*) and in enterocytes (*NP1>cdk4^R^*) and treated the flies with DSS. While control flies never showed chains of more than three small cells expressing Su(H)Gbe-lacZ, examples of larger chains or clusters of cells expressing Su(H)Gbe-lacZ could be found in ISC, but not in enterocyte knockdown strains, (Fig. S5), although at a low frequency compared to the *cdk4^3^* mutant flies, implying that there are contributions of Cdk4 in both progenitor cells and differentiated enterocytes to the full *cdk4^3^* null phenotype.

Finally, non-damaged *cdk4^3^* mutant midguts also display similar examples, although at lower frequency, of clusters of cells with small nuclei and a variety of aberrant combinations of cell fate markers (Fig. 8C, arrows), indicating that these ISC/enteroblast mis-differentiation phenotypes occur during normal homeostasis and are enhanced, rather than caused, by DSS-induced damage.

**Fig. 9.**
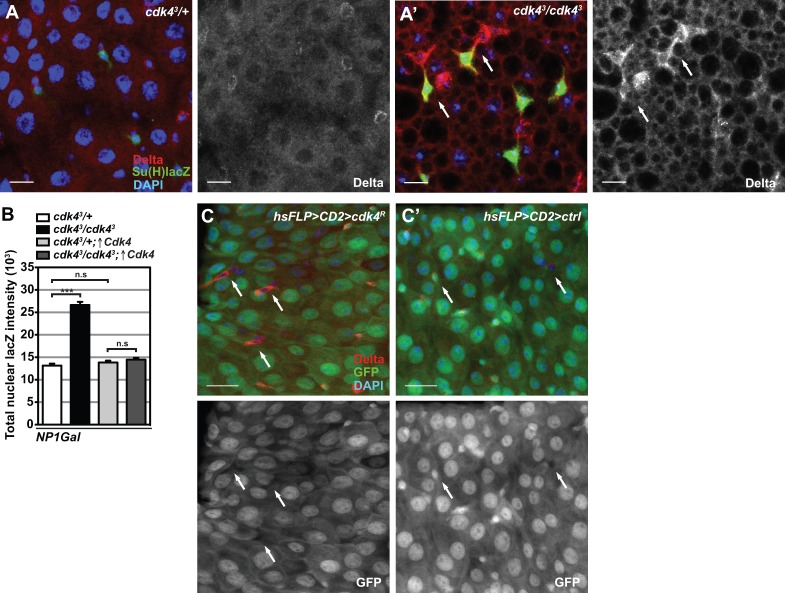
**Enterocyte growth status regulates Delta expression in wild type and *cdk4^3^* mutant ISCs.** (A,A′) Immunofluorescence staining against Notch reporter Su(H)Gbe-lacZ (Su(H)lacZ) (green) and anti-Delta (red). In A′ arrows mark adjacent cells doubly positive for Delta protein and Notch pathway activation in *cdk4^3^* mutant AMG epithelium. Genotypes: *Su(H)Gbe-lacZ;esgGFP-Gal4-cdk4^3^*/*+* (*cdk4^3^*/*+*) and *Su(H)Gbe-lacZ;esgGFP-Gal4-cdk4^3^*/*cdk4^3^* (*cdk4^3^*/*cdk4^3^*). (B) Mean Dl-lacZ fluorescence signal per ISC nuclear area in AMGs with *NP1-Gal4*-driven expression of Cdk4 in *cdk4^3^* mutants or controls. Genotypes: *NP1-Gal4-cdk4^3^/+;Dl-lacZ* (*cdk4^3^*/+), *NP1-Gal4-cdk4^3^/cdk4^3^;Dl-lacZ* (*cdk4^3^/cdk4^3^*), *NP1-Gal4-cdk4^3^/+;UAS-Cdk4/Dl-lacZ* (*cdk4^3^/+*; ↑*Cdk4*), *NP1-Gal4-cdk4^3^/cdk4^3^;UAS-Cdk4/Dl-lacZ* (*cdk4^3^/cdk4^3^*; ↑*Cdk4*). (C,C′) Flipout *Cdk4* knockdown clones. Clones are marked by presence of GFP and arrows show Delta-expressing and GFP negative wild-type clones in an otherwise RNAi epithelium. Flipout clones induced in a control background are shown in C′. Genotypes: *hsFLP,UAS-GFP*;*Act>CD2>Gal4,UAS-GFP;UAS-Cdk4^dsRNAi^* (*hsFLP>CD2>cdk4^R^*) and *hsFLP,UAS-GFP*;*Act>CD2>Gal4,UAS-GFP;UAS-lacZ* (*hsFLP>CD2>ctrl*). Scale bars: 10 µm. Mean+s.e.m;***P<0.001; n.s., not significant.

Varying the intensity of Delta activation in the ISC prior to cell division determines the levels of Notch pathway activation in the enteroblast daughter cell, specifying if this cell will become an enteroendocrine cell or an enterocyte. The Notch pathway is normally inactive in the ISC and inappropriate activation of Notch in this cell type leads to reduced self-renewal and terminal differentiation (Micchelli and Perrimon, 2006; Ohlstein and Spradling, 2007). Delta protein is normally partially inherited by the enteroblast after cell division and is thereafter rapidly removed and down-regulated, in order to ensure correct Notch activation in the enteroblast and prevent activation in the ISC (Ohlstein and Spradling, 2007). To ask whether alterations in Delta-Notch signalling might contribute to mis-differentiation in *cdk4^3^* mutants, we analysed levels of the Notch ligand Delta and employed the reporter Su(H)Gbe-lacZ, to monitor Notch pathway activity. Small cells in *cdk4^3^* mutant AMGs exhibited much higher Delta protein expression levels than cells in control guts (Fig. 9A,A′). Additionally, mutant guts displayed the aberrant occurrence of two adjacent small cells that both display strong Delta protein abundance with one of the cells in addition exhibiting elevated Notch pathway activation (Fig. 9A′, arrows). In mutant midguts the enteroblast appears to have either inappropriately retained Delta expression after mitosis or the mutant ISC has gained Notch activation. As varied levels of Delta protein in ISCs determine the extent of Notch activation in enteroblasts and succeeding cell fate determination, the DI-lacZ reporter transgene was used as a read-out of *Delta* transcriptional activation. Consistent with the immunofluorescence staining for Delta protein abundance, small cells residing in *cdk4^3^* mutant guts exhibit a large transcriptional activation of the *Delta* gene compared to control ISCs and re-expression of Cdk4 in *cdk4^3^* mutant enterocytes using *NP1-Gal4* as a driver entirely repressed this transcriptional increase (Fig. 9B). Random Flipout *Cdk4*-RNAi clones were produced by heat-shock and examined after 12 days. Heat-shock induction rendered nearly one hundred percent enterocyte clones with *Cdk4* knockdown (*hsFLP>CD2>cdk4^R^*), surrounding a mixture of GFP− wild-type ISC clones and GFP+ knockdown ISC clones. Multiple wild-type ISC clones displayed a clear increase in Delta protein levels (Fig. 9C, arrows) compared to clones induced in control intestines without *Cdk4* knockdown (*hsFLP>CD2>ctrl*) (Fig. 9C′, arrows). These results collectively demonstrate that *Delta* transcriptional activation and high protein abundance in *cdk4^3^* mutant and wild-type ISCs is a consequence of Cdk4-dependent functions in the surrounding enterocytes.

To exclude that Delta accumulation is a secondary consequence of excessively stressed mutant guts, multiple read-outs of stress were used (Biteau et al., 2008; Jiang et al., 2009; Zhou et al., 2013). There was no noticeable increase in capase 3 staining (data not shown), neither was there any activation of the JNK pathway as shown by microscopic examination of the reporter gene *puckered* (*puc*, pucE69) (Fig. S4D) and *puc* gene expression by qRT-PCR (Fig. S4E). Additionally, there was no activation of the JAK/STAT pathway (Fig. S4F).

In summary, these results demonstrate that *cdk4^3^* midguts do not show strong signs of stress or damage and we conclude that a primary consequence of Cdk4 deficiency in enterocytes in the midgut epithelium is an increase in protein abundance of Delta in ISCs and Delta retention in enteroblasts. This may potentially contribute to the observed alterations in ISC proliferation and differentiation.

## DISCUSSION

We have characterised the intestinal phenotypes of adult flies lacking the growth regulator Cdk4. We show that midguts mutant for Cdk4 display a sparse cellular appearance due to multiple causes including enterocyte endoreplication and growth defects, greatly reduced abundance of differentiated enterocytes and enteroendocrine cells, lower numbers of ISCs and enteroblasts and ISC/enteroblast differentiation defects. These phenotypes arise due to a combination of cell autonomous and cell non-autonomous functions of the CycD/Cdk4 complex in different cell types in the gut. Moreover, we have identified that enterocyte growth status in general acts to regulate ISC proliferation and differentiation.

Loss of Cdk4 function cell autonomously impairs enterocyte endoreplication and over-expression of CycD/Cdk4 causes kinase-dependent excessive endoreplication and growth of enterocytes. This is consistent with previous studies showing that loss of Cdk4 or CycD leads to impaired cellular growth and that ectopic expression of CycD/Cdk4 strongly induces endoreplication of post-mitotic tissues such as the larval salivary gland and the fat body (Frei et al., 2005; Meyer et al., 2000). The numbers of esgGFP+ ISCs/enteroblasts and numbers of Dl-lacZ expressing ISCs are reduced in *cdk4^3^* flies, which eclose with 30% fewer ISCs than control flies, demonstrating that Cdk4 is important for gut development during pupariation. Importantly the ISCs show a drastically decreased rate of turnover during maturation of adult flies, resulting in a 55% reduction in ISC numbers in 3-day-old flies that is maintained throughout the life of the animal. One drawback of our Cdk4 re-expression experiments is that all described experiments with Gal4 drivers (*Dl*, *esgGFP*, *NP1*) are non-conditional and Cdk4 is continuously expressed during larval stages, pupariation and adulthood. Unfortunately it was not possible to employ the temperature sensitive *tubulin-Gal80* conditional driver to attempt to uncouple the developmental and adult requirements for Cdk4 due to the fact that *cdk4^3^* or *cycd^1^* homozygous mutant flies are not viable when pupariation takes place at 18°C.

The dramatic reduction of the functioning stem cell compartment of the AMG in mature adults partly reflects a cell autonomous requirement for Cdk4 in proliferation of ISCs as shown by *Dl-Gal4*-driven rescue and RNAi knockdown experiments. Driving RNAi expression in both ISCs and enteroblasts in wild type flies using *esgGFP-Gal4* more closely approximated the *cdk4^3^* mutant phenotypes with respect to ISC, enterocyte and enteroendocrine cell numbers, and ISC/enteroblast-specific reintroduction of Cdk4 caused a stronger rescue of these mutant phenotypes than the ISC-specific rescue. However, since *esgGFP-Gal4* driven expression of transgenes can persist in early enterocytes (Shaw et al., 2010), reintroduction of Cdk4 in ISCs and enteroblasts also partially rescued enterocyte endoreplication defects, complicating the interpretation of this experiment. Importantly, enterocyte-specific reintroduction of Cdk4 using the *NP1-Gal4* driver impressively rescued ISC proliferation and abundance and partially rescued numbers of enterocytes and enteroendocrine cells, arguing that the growth status of enterocytes regulates ISC proliferation and differentiation. A series of experiments involving modulation of enterocyte growth through genetic manipulations of CycD/Cdk4, Rheb and Tsc1/Tsc2 further demonstrated that there is an intimate connection between enterocyte growth status and regulation of ISC abundance. Finally, MARCM stem cell clones expressing *Cdk4* RNAi showed defects in clonal expansion, consistent with our other findings of cell autonomous requirements for Cdk4 for ISC proliferation, enteroblast differentiation and cell non-autonomous roles of Cdk4 in enterocytes that influence progenitor cell functions.

**Fig. 10.**
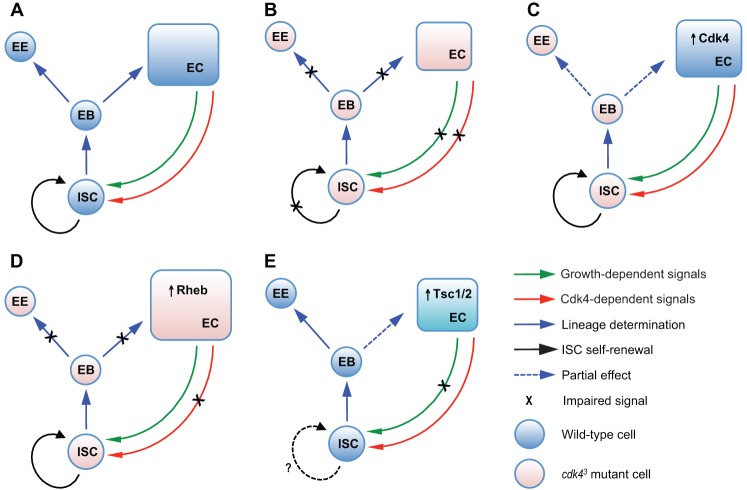
**Summary of Cdk4 activities in various AMG cell types.** (A) Schematic description of ISC self-renewal and lineage determination in wild-type AMGs showing the separate Cdk4-dependent (red arrow) and growth-dependent (green arrow) signals from the enterocytes (EC) to the ISCs that influence proliferation of ISCs (black arrow) and subsequent differentiation (blue arrows) of daughter cells. (B) In *cdk4^3^* mutant AMGs, crossed out lines represent loss of growth-dependent and Cdk4-dependent signalling, loss of ISC self-renewal and blockage of enteroblast (EB) differentiation to enterocytes and enteroendocrine cells (EE). (C) enterocyte-specific *Cdk4* expression in *cdk4^3^* AMGs restores Cdk4-dependent and growth-dependent signalling from enterocytes that results in normal proliferation of *cdk4^3^* ISCs and partial (blue dotted arrows) restoration of total ISC and enterocyte/enteroendocrine cell numbers. (D) enterocyte-specific Rheb expression in *cdk4^3^* AMGs restores enterocyte growth-dependent but not Cdk4-dependent signals, inducing self-renewal of ISCs without restoring enteroblast differentiation and numbers of enterocytes and enteroendocrine cells. (E) Repression of enterocyte growth by Tsc1/Tsc2 over-expression causes loss of growth-dependent but not Cdk4-dependent signals, resulting in reduced ISC abundance and a small decrease in numbers of enterocytes.

The failure of ISCs in *cdk4^3^* guts to proliferate and increase the number of differentiated enterocytes and enteroendocrine cells appears to be at least partly due to mis-differentiation and loss of stemness. These phenotypes also have cell autonomous and cell non-autonomous components. The demonstration that driving *Cdk4* RNAi in ISCs or in enteroblasts in wild type flies caused a large reduction in enteroendocrine cell numbers but only a very small reduction in enterocyte numbers argues that there is a cell autonomous requirement for Cdk4 in these cell types to regulate the fate decision of enteroblasts to form enteroendocrine cells. Uncovering the mechanism underlying this effect will require further study. In this context a recent study showed that bidirectional Notch signalling between ISCs and progeny cells is important for cell fate determination and for maintenance of ISC multipotency (Guo and Ohlstein, 2015), providing a potential starting point to investigate how Cdk4 function in enteroblasts can regulate enteroendocrine cell formation. Intriguingly, restoring Cdk4 expression in enterocytes largely rescued the reduced numbers of enterocytes and enteroendocrine cells in *cdk4^3^* mutant guts, and enterocyte-specific *Cdk4* knockdown in wild type guts reduced total numbers of enterocytes and enteroendocrine cells, implying that correct differentiation of enteroblasts to generate mature intestinal cells also requires Cdk4 function in enterocytes. This cell non-autonomous requirement for Cdk4 in enterocytes in controlling differentiation of ISCs/enteroblasts can be at least partially uncoupled from its function in regulating enterocyte growth since enterocyte-specific Rheb over-expression in *cdk4^3^* mutant guts restored enterocyte growth but did not rescue the numbers of enterocytes and enteroendocrine cells, despite initially increasing ISC proliferation. This argues that enterocytes control ISC proliferation and enteroblast differentiation through at least two separate cell non-autonomous processes; one dependent on enterocyte growth status and one dependent on the presence of Cdk4 in enterocytes. Fig. 10 schematically summarises these enterocyte growth status-dependent and Cdk4-dependent cell non-autonomous regulatory functions in the context of the different genotypes that were employed in this study.

Molecular analyses of markers of ISCs and enteroblasts support the hypothesis that mis-differentiation contributes to the reduced numbers of ISCs and differentiated cells in *cdk4^3^* mutant AMGs. Both unchallenged and DSS-treated *cdk4^3^* mutant guts display aberrant clusters of small cells in which several adjacent cells are doubly positive for the Notch reporter Su(H)Gbe-lacZ and for esgGFP*,* marking them as enteroblasts, while other cells in the clusters either express Su(H)Gbe-lacZ but do not express esgGFP, or express neither of these markers, suggestive of a loss of stemness or correct cell identity.

These phenotypes may potentially be at least partly reconciled by our observation that an as-yet-unidentified Cdk4 function in enterocytes acts cell non-autonomously to cause *Delta* transcription and protein abundance to be highly up-regulated in small cells in both wild type and *cdk4^3^* mutant AMGs. Increased Delta abundance in ISCs typically occurs as a consequence of enterocyte cell death or ablation, leading to strong Notch pathway activation in enteroblasts and concurrently results in new enterocytes being formed (Biteau et al., 2008; Jiang et al., 2009). On the other hand, low Delta expression in ISCs causes weaker Notch activation in enteroblasts and thus differentiation towards an enteroendocrine cell fate. Importantly, correct lineage determination requires that Delta protein inherited by the enteroblast from the ISC is rapidly down-regulated after cell division, ensuring that the Notch pathway remains inactive in the ISC (Ohlstein and Spradling, 2007). It is plausible that strong Delta activation in *cdk4^3^* ISCs could lead to excessive deposition of Delta protein in enteroblasts after cell division and thus strong Notch pathway activation in both the ISC and enteroblast. Notch activation in ISCs is known to cause loss of stemness and self-renewal properties of the ISC (Biteau et al., 2008), consistent with our observations of low numbers of ISCs that do not increase over the life span of the fly. Furthermore, simultaneous Notch activation and Delta retention in enteroblasts causes loss of differentiation (Biteau et al., 2008), also consistent with our observations of fewer differentiated enterocytes and enteroendocrine cells.

Our findings contribute to an increasing awareness that ISC proliferation and differentiation are regulated by both cell autonomous and cell non-autonomous mechanisms and support the idea that enterocytes contribute to a midgut stem cell niche. A stem cell niche is defined as a restricted position in an organ that supports the self-renewing division of stem cells, additionally preventing them from differentiating (Lin, 2002). While a permanent stem cell niche has not been defined in the *Drosophila* intestine, paracrine Wingless signalling from the intestinal muscles controls self-renewal of ISCs (Lin et al., 2008) and there is a transient stem cell niche in the larval gut (Mathur et al., 2010). Additionally, contact between ISCs and enteroblasts can regulate ISC proliferation in the midgut (Choi et al., 2011). Contrary to the conclusions made in previous studies arguing that lineage selection and differentiation is an intrinsic function relying entirely on the ISCs themselves (Casali and Batlle, 2009), we show that ISC proliferation is dependent on the growth status of surrounding enterocytes and that ISC/enteroblast differentiation is dependent on an as-yet-uncharacterised function of Cdk4 in enterocytes as well as on a general dependency on enterocyte growth status. These dependencies could potentially function via requirements for essential cell-cell interactions. In this context, it is interesting that the expression levels of several proteins that mediate cell-cell interactions, including Armadillo (adherens junctions) and Discs large (septate junctions) are altered in *cdk4^3^* mutant guts (our unpublished observations). Alternatively, or additionally, we speculate that growth impaired enterocytes may potentially not release sufficient ligands that activate the Wnt, Jak/Stat, EGFR, JNK or insulin signalling pathways, which are known to promote ISC proliferation and influence differentiation (Jiang and Edgar, 2011). While further studies are required to unravel these mechanisms, our observation that enterocyte growth status influences differentiation of ISC daughter cells and overall gut homeostasis provides further evidence that the cellular microenvironment is an essential regulator of stem and progenitor cell behaviours.

## MATERIALS AND METHODS

### Fly maintenance and stocks

Experimental flies were kept on standard food under non-crowded conditions and changed on fresh food every two days. Only male adults were used in this study. Cell counting and analyses were performed on the anterior midgut region (anterior to the acidic region). For lipid and glycogen quantifications, including the starvation assay, a maximum of 50 larvae were allowed to develop per vial. Adult flies were kept in vials at a density of 15-40 animals and were changed on fresh food every two days.

Wild type and mutant stocks: Lines *yw* and *w^iso^* were used as wild type controls. Mutant null allele stocks used: *yw;cdk4^3^*/*CyO-GFP* (Meyer et al., 2000) and *cycd^1^/FM7-GFP* (Emmerich et al., 2004), *yw;cdk4^3^*/*CyO-GFP;UAS-Rheb/TM6B*, *yw;cdk4^3^*/*CyO-GFP;Dl^05151^-lacZ/TM6B*, *yw;cdk4^3^*/*CyO-GFP;Dl^05151^-Gal4/TM6B*, *yw;NP1-Gal4-cdk4^3^*/*CyO-GFP;Dl^05151^-lacZ/TM6B, Su(H)Gbe-lacZ;esg-Gal4, UAS-GFP-cdk4^3^*/*CyO-GFP*, *yw;cdk4^3^/CyO-GFP;UAS-Cdk4*, *yw;cdk4^3^/CyO-GFP;UAS-Cdk4^D175N^*. *UAS-Cdk4 III.2* and *UAS-Cdk4^D175N^*-*mycIII.4* were both originally published in Meyer et al. (2000). All *cdk4^3^* and RNAi experiments were done in an *yw* genetic background while all *cycd^1^* and over-expression experiments were done in a *w^iso^* background.

Gal4 drivers: For all experiments with Gal4 drivers, the driver combined with a wild-type chromosome in either *yw* or *w^iso^* background was used as control. *yw;;Dl^05151^-Gal4*/*TM6B* (ISC expression), *w;;Su(H)Gbe-Gal4/TM3* (enteroblast expression), *yw;esg-Gal4,UAS-lacZ, UAS-GFP/CyO* (ISC/enteroblast expression), *yw;NP1-Gal4/CyO-GFP* (enterocyte expression) and *yw;NP1-Gal4*/*CyO;Dl-lacZ/TM6B.* For *cdk4^3^* experiments *esg-Gal4* and *NP1-Gal4* were recombined on the same chromosome as the *cdk4^3^* allele (stocks: *yw;NP1-Gal4-cdk4^3^/CyO-GFP* and *yw;esgGal4, UAS-GFP-cdk4^3^/CyO-GFP*).

For CycD/Cdk4 over-expression and Cdk4-RNAi clone induction the following line was used: *hsFLP^122^;;Act>CD2>Gal4, UAS-GFP*.

Reporters: *yw;;Dl^05151^-lacZ/TM6B* (Beebe et al., 2010; Zeng et al., 2010), *Su(H)Gbe-lacZ;;* (Ohlstein and Spradling, 2007), *yw;cdk4^3^*/*CyO-GFP;Stat10xGFP/TM6B*, *yw;cdk4^3^*/*CyO-GFP;UAS-pucE69/TM6B* and *Su(H)Gbe-lacZ;cdk4^3^*/*CyO-GFP*.

RNAi lines: *yw;UAS-Cdk4^dsRNAi^/CyO-GFP*, *yw;;UAS-Cdk4^dsRNAi^/TM6B* and *yw;UAS-Cdk4^dsRNAi^;Dl^05151^-lacZ.* Two different *UAS-Cdk4^dsRNAi^* lines were used, both produced the same phenotypes (stock numbers #40576 and #40577, Vienna *Drosophila* RNAi Center, IMP, Vienna, Austria). Experimental RNAi adults were reared and kept at 25°C throughout their entire lifespan.

UAS transgenes: *yw;UAS-Tsc1, UAS-Tsc2/CyO* (Tapon et al., 2001), *yw;;UAS-Rheb/TM6B* (Stocker et al., 2003), *w;UAS-Cdk4^D175N^-mycIII.4, UAS-CycD* and *w;;UAS-Cdk4 III-1* (Meyer et al., 2000), *w;;UAS-Cdk4-myc III.4, UAS-CycD III.1* (Datar et al., 2000).

### MARCM experiments

For induction of MARCM clones the following stocks were used: *yw hsFLP, UAS-GFP;;tubGal4, FRT82, tubGal80/TM6B*, *yw;;FRT82/TM6B* and *yw;UAS-Cdk4^dsRNAi^/CyO-GFP;FRT82/TM6B.* Experimental animals developed at 25°C and were collected immediately after eclosion and aged. At three days of age the adults were heat-shocked at 37°C for 1 h for three consecutive days and analysed either three days or 32 days after the last heat-shock. The experimental flies were kept at 18°C after the first heat-shock until the time of analysis.

### Antibody stainings for immunofluorescence

Flies were dissected in PBS, fixed in 4% paraformaldehyde (PFA)/PBS (Electron Microscopy Sciences) for 45 min and washed in 0.2% Triton X-100/PBS (PBST). Samples were washed in increasing concentrations of PBST (0.5% PBST 20 min, 1% PBST 1 h), followed by blocking with 0.2% BSA for 2 h at room temperature or overnight at 4°C. After blocking, samples were washed down to 0.2% PBST (1% PBST, 0.5% PBST for 20 min each) and incubated with primary antibody in 0.2% PBST 2-3 h at room temperature or overnight at 4°C, followed by washing in 0.2% PBST for 2-3 h. Samples were incubated with secondary antibody for 3 h at room temperature or overnight at 4°C and incubated with DAPI (0.5 mg/ml stock solution, 1:1000 dilution, Molecular Probes) for 5 min, washed 2-3 h in 0.2% PBST, rinsed in 1× PBS for 5 min, mounted on slides and covered with Vectashield mounting medium (Vector Labs). All steps were done with mechanical agitation. Primary antibodies used: monoclonal anti-mouse Delta (1:100, DSHB), monoclonal anti-mouse prospero (1:100, DSHB), monoclonal anti-mouse Armadillo (1:50, DSHB), monoclonal anti-mouse Lamin (1:100, DSHB), polyclonal anti-rabbit β-gal (1:500, Promega). Secondary antibodies used: goat anti-mouse Alexa Fluor 568 and 488 (Invitrogen) and anti-rabbit Alexa Fluor 568 and 488 (Invitrogen) at dilutions between 1:500 and 1:1000.

### Image acquisition, processing and analysis

Images of immunofluorescently stained adult intestines were acquired using either a Leica SP2-AOBS or a Leica SP5 Resonant-APD laser scanning confocal microscope with a 40× or 63× Oil Objective. Serial *Z*-sections were taken with a distance between 0.2-0.4 µm and average intensity projections of all sections were created either with Leica Confocal Software or Fiji. Images were processed using Photoshop (Adobe) and the colour intensity of images was enhanced equally for all images within the same experiment using linear adjustment. Quantifications of nuclear areas were done in ImageJ (NIH) and are shown in µm^2^. Results are shown as mean with either standard deviation (s.d.) or standard error of mean (s.e.m.).

### Quantification of DAPI and lacZ fluorescence intensities

Adult intestines were dissected, stained using DAPI and imaged with a Leica SP5 Resonant-APD laser scanning confocal microscope, using identical settings for laser power including excitation/emission range and background for each fluorochrome and across all intestines and genotypes within the same experiment. Average projections of stacks were created and analysed in Fiji as greyscale. Enterocyte nuclear area was manually circumscribed and the size and the fluorescence intensity were determined. The relative DNA content is reflected by the integrated density, which is the product of the area and the average fluorescence (expressed as mean grey value) (Boudolf et al., 2004; Maes et al., 2008). The background fluorescence was recorded separately for each image and subtracted from the obtained intensity values. The same method was used for measuring lacZ intensity in ISCs after staining with an anti-lacZ antibody.

### Quantifications of ISC, enteroblast, enterocyte and enteroendocrine cell numbers

Enterocyte numbers were quantified based on confocal images of the entire anterior midgut region of 3-5 midguts from 7-8-day-old males per genotype. Serial sections were acquired across the entire thickness of the intestinal epithelium and average projections were created separately for the dorsal and ventral epithelial layer. Cells with a diameter of at least 16 µm were counted as enterocytes. Quantifications of ISC, enteroblast and enteroendocrine cell numbers were done using a Scope A1 microscope (Zeiss) and for each genotype 6-15 intestines were counted. Counting of cells was performed across different adult ages as specified for ISCs/enteroblasts and in mature (7-12 days old) adult flies for enteroendocrine cells. Labelled cells were quantified across the entire anterior midgut (prior to the acidic region) and the dorsal and ventral epithelial layers were counted separately. The numbers were tallied to give total cell numbers per anterior midgut region. ISC were marked by presence of lacZ, ISCs and enteroblasts were labelled with GFP and enteroendocrine cells were marked with prospero. Results are shown as mean and standard deviation (mean+s.d.).

### BrdU incorporation assay

Adult males aged either 2 or 6 days were fed standard fly food supplemented with 5-bromo-2-deoxyuridine (BrdU, final concentration 0.2 mg/ml, Calbiochem) for 48 h. The flies were dissected and fixed in 4% PFA for 45 min and treated with 3 M HCl for 30 min. After HCl denaturation standard staining protocol was used and the guts were stained using monoclonal rat anti-BrdU antibody (1:1000 dilution, Oxford Biosystems). Anti-rat Alexa Fluor 488 was used as secondary antibody (1:5000, Invitrogen).

### DSS feeding

Aged mature males (7-10 days old) were fed standard fly food supplemented with Dextran Sodium Sulfate (DSS) salt reagent (final concentration 3%, MP Biomedicals). The DSS feeding was done at 25°C and the flies were fed for 48-72 h with the plates being changed daily.

### Lipid quantification

Ten to fifteen adult flies (per biological replica) were shock-frozen in liquid nitrogen and homogenized in 0.05% Tween/PBS using a MM2 homogenizing machine (Retsch). Samples and standards were heated to 70°C for 5 min to inactivate all enzymes. Samples were added un-centrifuged to 1 ml cuvettes containing 800 µl of Triglyceride Reagent (Sigma) and incubated at 37°C for 5 min, following initial absorbance reading at 540 nm. After addition of 200 μl Triglyceride lipase (Sigma), samples and standards were incubated at 37°C for 5 min and final absorbance was recorded. Measured data was interpolated from a standard curve and normalised per milligram total body weight. Results are shown as mean+s.d. from 3-5 biological replicates.

### Glycogen quantification

Ten to fifteen adult flies (per biological replica) were shock-frozen in liquid nitrogen and homogenized in 250 μl Na_2_CO_3_ (0.25 M) using a MM2 homogenizing machine (Retsch), following 2 h incubation at 95°C. After incubation 150 μl 1 M acetic acid was added together with 650 μl Na-acetate (0.2 M, pH 5.2) and samples were centrifuged at 15,700 ***g*** for 10 min. 900 μl of the supernatant was transferred to a new Eppendorf tube and centrifuged 15,700 ***g*** for 5 min. 100 μl of the supernatant was transferred to an Eppendorf tube where 2 μl of amyloglucosidase enzyme (67.4 U/mg, Sigma) was added, followed by an overnight incubation at 56°C. After incubation, glycogen in the samples is converted to glucose. Subsequently, 20 μl of each sample was added to 100 μl of Glucose Assay Reagent (Sigma) in a 96-well plate, incubated for 20 min at room temperature and absorbance was read at 340 nm using a SpectraMax 190 96-well spectrophotometer. For each 96-well plate measurement, a glycogen standard (glycogen from bovine liver type IX) was made and incubated together with respective samples with addition of amyloglucosidase enzyme. Measured data was interpolated from a standard curve and normalised per total milligram body weight. Results are shown as mean+s.d. from 3-5 biological replicates.

### Starvation assay

Starvation medium was made with 1× PBS mixed with agarose (1%) and poured into empty food vials. Flies in batches of 15-30 flies were used per biological replicate; at least three biological replicates were used per genotype. Starvation was always started in the afternoon and the numbers of dead/alive flies were recorded at 1-h intervals until all the flies were dead. Data is shown as mean+s.d.

### RNA extraction and qRT-PCR

For RNA extraction 30-40 male adult intestines for each genotype were dissected in 1× PBS, with the hindgut removed, and transferred to RNAlater (Sigma). Tissues were lysed with TriZol (Sigma) and subsequent RNA extraction was performed using RNA Extraction Kit (Macherey-Nagel) following the manufacturers recommendations. cDNA synthesis was done with Ready-To-Go-Prime-Beads (GE Healthcare) following the manufacturers recommendations. Quantitative real-time PCR was performed using Roche SYBR Green Mix and using the Light Cycler 480 (Roche). All experiments were done in at least biological triplicates and the results are shown as mean+s.d. The ribosomal gene *rp49* was used as internal control.

### Clonal analysis

Fly stocks were crossed to generate offspring with the following genotypes: *yw* *hsFLP,UAS-GFP;UAS-lacZ;Act>CD2>Gal4,UAS-GFP*, *yw* *hsFLP,UAS-GFP;UAS-Cdk4^dsRNAi^;Act>CD2>Gal4, UAS-GFP* and *yw* *hsFLP,UAS-GFP; UAS-CycD, UAS-Cdk4;Act>CD2>Gal4, UAS-GFP*. For induction of 100% *Cdk4^dsRNAi^* ‘flip-out’ clones globally across the intestine adult males were heat-shocked at 37°C for 40 min at three days post eclosion and dissected 12 days post heat-shock (Fig. 7). Single Cdk4 knockdown and CyclinD/Cdk4 overexpression clones were induced spontaneously at 25°C without additional heat-shock (Fig. 1).

### Statistical analysis

All statistical significances were calculated in Prism 5 for Mac OS X (GraphPad Software, Inc) using unpaired Student's *t*-test (two tailed, two sample equal variance). *P<0.05, **P<0.01 and ***P<0.001; n.s., not significant. For survival curves the Log-rank test was performed.
